# Ultra-High-Expansion-Ratio Nanocellular Semicrystalline Polymer Foams: Preparation of Chain-Extender-Mediated PLA/PPC Foams via Supercritical CO_2_

**DOI:** 10.3390/polym18182273

**Published:** 2026-09-17

**Authors:** Xubo Zhao, Xuejun Liu, Fengrui Hu, Hongfu Zhou, Yafeng Deng

**Affiliations:** 1 Key Laboratory of Processing and Application of Polymeric Foams of China National Light Industry Council, School of Advanced Materials and Future Technology, Beijing Technology and Business University, Beijing 100048, China; 2431042106@st.btbu.edu.cn (X.Z.); 17507561319@163.com (F.H.); zhouhongfu@th.btbu.edu.cn (H.Z.); 2 School of Computer and Artificial Intelligence, Beijing Technology and Business University, Beijing 100048, China; dengyafeng@th.btbu.edu.cn

**Keywords:** Poly(lactic acid), Poly(propylene carbonate), nanocellular structure, chain-extension modification, bio-based and biodegradable foams

## Abstract

The inherent brittleness, retarded crystallization kinetics and insufficient melt strength of polylactic acid (PLA) severely limit its applications in foaming. This study proposed a reactive chain extender and compatibilizer strategy, which involved introducing the multifunctional epoxy-based chain extender KL-E4370 (0.6 wt%) into a PLA/polypropylene carbonate (PPC) blend system to simultaneously enhance melt strength and interfacial compatibility, with the aim of preparing high-expansion-ratio nano-cellular foams by means of the physical foaming assisted by supercritical CO_2_. The impact of PPC loading and foaming temperature on the crystallization behavior, rheological properties, cell morphology, and compressive properties of the blends were systematically investigated. As a result, chemical interactions occurred between the chain extender and terminal carboxyl and hydroxyl moieties of PLA and PPC during ring-opening reactions, which produced a PLA–chain extender–PPC copolymer in situ. This significantly improved melt elasticity, reduced interfacial tension, and promoted the even distribution of the PPC phase. At the same time, the addition of PPC enhanced the CO_2_ adsorption capacity of PLA. The PLA/PPC15-CE0.6 formulation exhibited the highest crystallinity (5.9%) and a refined spherulite morphology, providing abundant interfaces between crystalline and amorphous regions that serve as auxiliary nucleation sites. At the optimal foaming temperature of 115 °C, this formulation achieved an excellent foaming volume expansion ratio of 7.3 times, an average cell size of 660 nm, and a maximum cell density of 1.1 × 10^13^ cells/cm^3^. With further elevation of the foaming temperature to 117 °C and beyond, excessive softening of the matrix triggered cell coalescence, causing the cell sizes to increase sharply beyond the nanoscale range and the cell density to drop significantly. Samples foamed at 115 °C retained a compressive strength of 2.7 MPa under 70% strain, demonstrating a good balance between lightweight properties and mechanical integrity. This study provides a systematic mechanism understanding and operational directions for the sustainable fabrication of high-performance bio-based biodegradable polymer nanoporous materials.

## 1. Introduction

Polymer nanofoams are a class of porous materials featured by cell sizes smaller than 1 μm and cell density exceeding 10^12^ cells/cm^3^ [1]. Compared to traditional microporous foams (with cell sizes ranging from 10 to 100 μm and cell density smaller than 10^12^ cells/cm^3^), nanofoams, due to their unique size effects and interfacial synergy mechanisms, exhibit superior thermal insulation, sound insulation, and mechanical properties while maintaining their lightweight characteristics [2]. When cell sizes are reduced to the nanoscale, the thickness of the cell walls correspondingly decreases, allowing the nanocells to act as effective strain concentrators, thereby significantly improving the toughness and impact strength of the foam [3]. Research indicates that the thermal conductivity of nanofoam can be as low as 0.09 W/(m·K) or less, offering broad application prospects in fields such as building insulation, cold-chain logistics, aerospace, and medical devices [4,5,6,7,8]. Furthermore, the high specific surface area and porous structure of nanofoam endow it with excellent adsorption properties and drug-release capabilities, demonstrating unique advantages in applications such as biological tissue engineering scaffolds and functional separation membranes [9].

Polylactic acid (PLA) is an aliphatic polyester synthesized from renewable biomass resources such as corn and sugarcane [10]. It possesses excellent biodegradability, high strength, and high modulus, and is regarded as an ideal green material to replace traditional petroleum-based plastics [11,12]. With the introduction of global “plastic restriction” and “plastic ban” policies, the market demand for PLA is growing rapidly. The large-scale promotion and application of PLA hold significant strategic importance for alleviating global plastic pollution, reducing dependence on fossil resources, and achieving carbon neutrality goals [13]. However, the inherent brittleness of PLA, its slow crystallization kinetics, and its extremely low melt strength severely limit its application in the field of foamed materials [14,15,16]. The weak solubility of CO_2_ in pure PLA during supercritical carbon dioxide (scCO_2_) foaming, along with low nucleation efficiency and restricted cell growth, makes it difficult to obtain high-volume expansion ratio (VER) foams with uniform cell structures [17]. This has become one of the core bottlenecks constraining the industrialization of PLA foaming materials.

ScCO_2_-mediated physical foaming represents an eco-friendly and highly efficient process that possesses prominent merits, including simple operation, no residual organic solvents, and the recyclability of the foaming agent [18,19]. The critical temperature (31.1 °C) and critical pressure (7.38 MPa) of scCO_2_ are mild, making it feasible to implement in industrial production. Compared to chemical foaming methods, scCO_2_ physical foaming avoids the release of harmful chemical decomposition products, and the foaming agent can be completely removed from the polymer matrix, eliminating the risk of residual toxicity. More importantly, the dissolution and diffusion behavior of scCO_2_ in polymers can be finely controlled by adjusting temperature and pressure, facilitating effective regulation of the nucleation and growth processes of the bubbles. scCO_2_ also exhibits a significant plasticizing effect on PLA, effectively lowering the glass transition temperature and melt viscosity of the polymer, promoting the mobility of molecular segments, and providing a favorable local free-volume environment for bubble nucleation [17,20]. At the same time, dissolved CO_2_ molecules undergo phase separation due to supersaturation during rapid depressurization, forming a large number of gas nuclei within the polymer matrix. This heterogeneous nucleation mechanism helps increase cell density and refine cell size [21]. By adjusting process parameters such as saturation temperature, saturation pressure, and depressurization rate, systematic control over cell morphology can be achieved [22,23].

Although researchers have explored various methods such as blend modification, chain-extension modification, filler reinforcement, and crystallization control to enhance the melt strength and foaming properties of PLA, research on the preparation of high-VER PLA nanofoams remains limited [24,25,26,27]. Wang et al. [28] successfully prepared PLA nanofoam materials with a maximum cell density of 1.76 × 10^13^ cells/cm^3^ and a maximum VER of 3.71. Li et al. [29] used a styrene–acrylonitrile–allyl methacrylate terpolymer to modify PLA foam materials. As reported, these foam materials exhibited a minimum average cell size of 249 nm, and the average cell density increased to 2.48 × 10^13^ cells/cm^3^, although the maximum VER was limited to 2.22. Among the aforementioned methods, chain-extension modification has attracted widespread attention due to its high efficiency and controllability. The epoxy-based chain extender KL-E4370 is a styrene–methacrylate glycidyl ester copolymer. The multiple epoxy functional groups on its molecular chain can undergo ring-opening reactions with the carboxyl (-COOH) and hydroxyl (-OH) groups at the ends of the PLA molecular chain, significantly increasing the polymer’s molecular weight, melt elasticity, and strength through chain extension and long-chain branching [30]. Research has shown that KL-E4370 can effectively suppress thermal degradation during PLA processing and improve its melt strength. The polydisperse viscosity, storage modulus, and strain hardening behavior of the chain-extended modified polymer are significantly enhanced, thereby effectively inhibiting cell coalescence and collapse during the foaming process. At the same time, the introduction of polypropylene carbonate (PPC) into a melt blend with PLA can toughen the PLA by leveraging the excellent toughness and good gas barrier properties of PPC [31,32]. Simultaneously, the introduction of PPC improved the solubility of CO_2_ in PLA [33,34,35]. However, PLA and PPC form thermodynamically immiscible blends. After simple blending, this system exhibits high interfacial tension and weak interfacial bonding, which severely degrades the mechanical properties of material and foaming uniformity. During melt blending, chain extenders can generate copolymers in situ at the PLA-PPC interface through reactive compatibilization, thereby reducing interfacial tension and strengthening interfacial bonding, thus providing an effective solution to the problem of phase incompatibility [21,23,36,37].

This study proposed the incorporation of the KL-E4370 chain extender into a PLA/PPC blend system to achieve reactive chain-extension modification via melt blending. This approach improves interfacial adhesion between the blended phases while enhancing the melt strength and foaming performance of the blend, and utilizes scCO_2_ physical foaming technology to prepare high-expansion-ratio PLA nanofoam. In this study, the PLA/PPC blend was chain-extended using 0.6 wt% KL-E4370 chain extender. The role of process parameters on the rheological behavior, thermal properties, and crystallization behavior of the blend was systematically investigated; a detailed analysis was conducted of the structure–property relationships between process parameters, such as saturation temperature, saturation pressure, foaming temperature and cell morphology (including cell size, cell density, and VER); and the formation mechanism of the nano-porous structure, as well as the synergistic mechanism of in situ volume expansion and reinforcement by the chain extender, was thoroughly explored. Through this study, we aim to provide a systematic theoretical basis and reliable technical reference for the green preparation of high-performance biodegradable polymer nanofoam materials.

## 2. Materials and Methods

### 2.1. Materials

The PLA with a D-content of 2%, obtained from Nature Works in the United States (Plymouth, MN, USA), has a density of 1.24 g/cm^3^ and a number-average molecular weight of approximately 100,000 g/mol, and is designated as grade 4032D. The PPC was purchased from the Huafeng Group (Wenzhou, China). Its grade is HF-901 with a number-average molecular weight of 200,000 g/mol. The chain extender CE (KL-E4370) was obtained from Shanxi Chemical Industry (Xi’an, China), and has a molecular weight of 6500–7200 and an epoxy equivalent of 280–310 g/mol (Figure S3). Carbon dioxide was purchased from Beijing Dongfang Medical Gas Co., Ltd. (Beijing, China), with a purity of 99.999%.

### 2.2. Preparation of Different PLA Specimens

Prior to the blending process, the PLA was dried continuously for 6 h in a vacuum desiccator at 80 °C. To prepare pure PLA and distinct PLA/PPC-CE specimens, a mixture of PLA and PPC was blended for 15 min at 180 °C using a torque rheometer at a speed of 60 rpm. Specimens were formulated with varying contents of PPC (5, 10 and 15 wt%) and KL-E4370 (0.6 wt%) (Table 1), and the corresponding specimens were denoted as PLA, CEPLA, PLA/PPC5, PLA/PPC5-CE0.6, PLA/PPC10-CE0.6 and PLA/PPC15-CE0.6, respectively. Based on preliminary test results (Figure S2 and Table S1), an addition level of 0.6 wt% KL-E4370 was selected. The tests showed that, at this concentration, the expansion ratio of the final foam sample was higher than that achieved at other concentrations. Subsequently, the resulting specimens were thermocompressed into 4 mm-thick sheets using a plate vulcanizer at 180 °C and 5 MPa. Afterwards, the sheets were cut into several 2 cm × 1 cm test specimens for use in subsequent experiments.

### 2.3. The Process of Preparing PLA Foam with Different Formulations

ScCO_2_ was used as a physical blowing agent. Various specimens were placed in a stainless steel high-pressure foaming reactor and foamed for 2 h at the pressure of 30 MPa and temperatures of 111, 113, 115, 117, and 119 °C. Subsequently, the pressure was rapidly released to obtain PLA foams with different VER. The pressure was rapidly released to atmospheric pressure within approximately 1–2 s by fully opening a high-speed ball valve, achieving an average depressurization rate of about 15–30 MPa/s.

### 2.4. Characterization Tests

#### 2.4.1. Differential Scanning Calorimetry (DSC)

To evaluate the thermodynamic behavior of different PLA specimens, DSC analysis was performed under a nitrogen atmosphere. First, each PLA specimen (approximately 5–10 mg) was heated to 200 °C to reach equilibrium and held isothermally for 3 min to eliminate the thermal history. Subsequently, the specimens was cooled to 40 °C at a rate of 10 °C/min. Upon reaching 40 °C, the specimens were held at that temperature for 3 min. Finally, they were reheated to 200 °C at the same heating rate. The crystallinity (χc) of each PLA specimens was calculated using the following formula [38]:

(1) $$\chi_{c}(\%)=\frac{\Delta H_{m}-\Delta H_{cc}}{\omega_{PLA}\times \Delta H_{m}^{0}}\times 100\%$$

in which $\Delta H_{m}$ and $\Delta H_{cc}$ represent the enthalpy of fusion and the enthalpy of cold crystallization for the specimen test, separately, while $\omega_{PLA}$ represents the mass fraction of PLA in each specimen. $\Delta H_{m}^{0}$ denotes the standard enthalpy of fusion for crystalline PLA, with a measured value of 93.7 J/g.

#### 2.4.2. Polarized Optical Microscopy (POM)

To observe the isothermal crystallization behavior of various PLA specimens at 130 °C, experiments were conducted using POM (59XF, Shanghai CSOIF Co., Ltd., Shanghai, China) at 130 °C. First, each PLA specimen was heated from 25 °C to 200 °C at a rate of 10 °C/min, held for 5 min to eliminate thermal history, and then cooled to 130 °C and maintained isothermally for 45 min to observe the changes in spherulitic morphology.

#### 2.4.3. Rheological Properties

To determine the rheomechanical parameters of various PLA specimens, a rotational rheometer (Ares Rheometer, TA, New Castle, DE, USA) was used. Measurements were performed on various PLA specimens using 25 mm parallel plates (with a gap of 1 mm), and the temperature was maintained at a constant 180 °C throughout the testing process. In addition, when configuring the initial test parameters, care should be taken to set the angular frequency (ω) (0.1~100 rad/s) and strain (1%), which made the rheological behavior locate in the linear viscoelastic region [39,40,41].

#### 2.4.4. Scanning Electron Microscopy (SEM)

SEM (Thermo Fisher Apreo 2s, Thermo Fisher, Waltham, MA, USA) was used to observe the microstructures of various specimens, including unfoamed and foamed specimens of various PLA types. The SEM was operated at a working voltage of 5 kV and a magnification of 5000×. Prior to SEM analysis, the fracture surfaces of various cryogenically quenched specimens were treated with Au sputter coating.

#### 2.4.5. Foaming Properties

After the foam morphology was observed with SEM, the cell density (“N”) was calculated in cells/cm^3^ using the following formula:

(2) $$N={\left[\frac{n}{A}\right]}^{\frac{3}{2}}\mathrm{VER}$$

in which n indicates the total number of cells observed in the SEM image at a specified magnification (5000×) and A (cm^2^) represents the area of the chosen observation region. The VER of various foams must be tested and calculated using Equation (3):

(3) $$\mathrm{VER}=\frac{\rho_{p}}{\rho_{f}}$$

where $\rho_{p}$ and $\rho_{f}$ indicate the measured densities of various PLA/PPC unfoamed and foamed specimens, respectively. All of the above density values were measured using a precision density balance (CPA2245, Sartorius, Göttingen, Germany) and are expressed in g/cm^3^. At least 5 SEM images were taken for each specimen, and the number of cells counted for different formulations and foaming conditions ranged from 100 to 150.

#### 2.4.6. Compressive Property Measurement

The compressive properties of various PLA foams were characterized using a universal testing machine (CMT6104, Shenzhen SANS Material Detection Co., Ltd., Shenzhen, China). In accordance with the GB/T 8813-2020 standard [42], the compressive strength and compressive modulus of each PLA foam at 70% relative deformation were determined at a constant compression rate of 1 mm/min. Specimens prepared under each foaming temperature condition must be tested at least five times.

#### 2.4.7. Fourier-Transform Infrared (FTIR) Spectroscopy

FTIR analysis was conducted using a MAGNA-IR560 spectrometer manufactured by Nicolet Instruments, Madison, WI, USA, to explore the chemical structure of the samples under investigation. Spectral data were captured over a wave-length range of 4000 to 400 cm^−1^, and 32 cumulative scans were performed for each measurement to ensure a reliable signal-to-noise ratio. Additionally, a background spectrum was automatically collected for each measurement and subsequently subtracted from the recorded data to improve the accuracy of the results. For specific results, please refer to the Supplementary Materials.

## 3. Results and Discussion

### 3.1. Analysis of Crystallization and Melting Behaviors of PLA/PPC5 and Different PLA/PPC-CE Specimens

**Figure 1:**
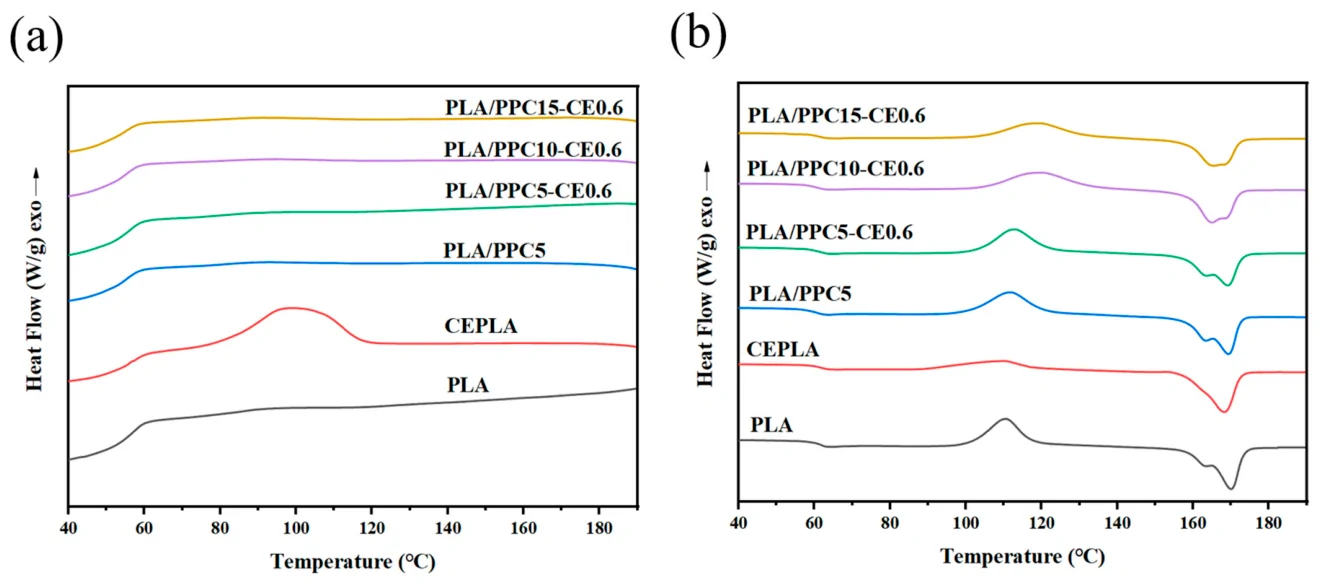
(**a**) Cooling and (**b**) heating curves of different PLA/PPC-CE specimens.

Figure 1 showed the DSC heating and cooling curves of PLA, CEPLA, and PLA/PPC-CE specimens at different PPC contents, and the corresponding thermal property parameters were summarized in Table 2. The heating curves reflected the glass transition, cold crystallization, and melting behavior, while the cooling curves revealed the crystallization behavior during the cooling of the melt.

In terms of glass transition temperature (T_g_), there were only minor differences among the samples; however, the introduction of a chain extender improved interfacial compatibility between the phases. Comparing the baseline specimen PLA/PPC5 (without CE0.6, T_g_ = 59.8 °C) with PLA/PPC5-CE0.6 (T_g_ = 60.1 °C), a slight increase in T_g_ was observed, indicating that the chain extender formed copolymer structures at the interface, which enhanced interfacial adhesion and restricted the segmental mobility of PLA. As the PPC content increased from 5 wt% to 15 wt%, the T_g_ of the specimens gradually decreased from 60.1 °C to 58.9 °C. This decrease in T_g_ could be attributed to two factors. First, the T_g_ of PPC itself was much lower than that of PLA; its addition was equivalent to introducing flexible segments into the PLA matrix, which increased the free volume and lowered the energy barrier for segment motion. Second, although the introduction of the chain extender KL-E4370 triggered a chain-extension reaction, the addition of PPC diluted the physical entanglement density between PLA chains, facilitating the movement of PLA molecular segments. It was worth noting that the decrease in T_g_ was relatively small for the three groups of specimens. This was attributed to the fact that the chain extender can enhance the compatibility between PLA and PPC, which formed a PLA–chain extender–PPC copolymer in situ, thereby limiting macroscopic phase separation between the two phases and preventing an excessive decrease in T_g_.

**Figure 2:**
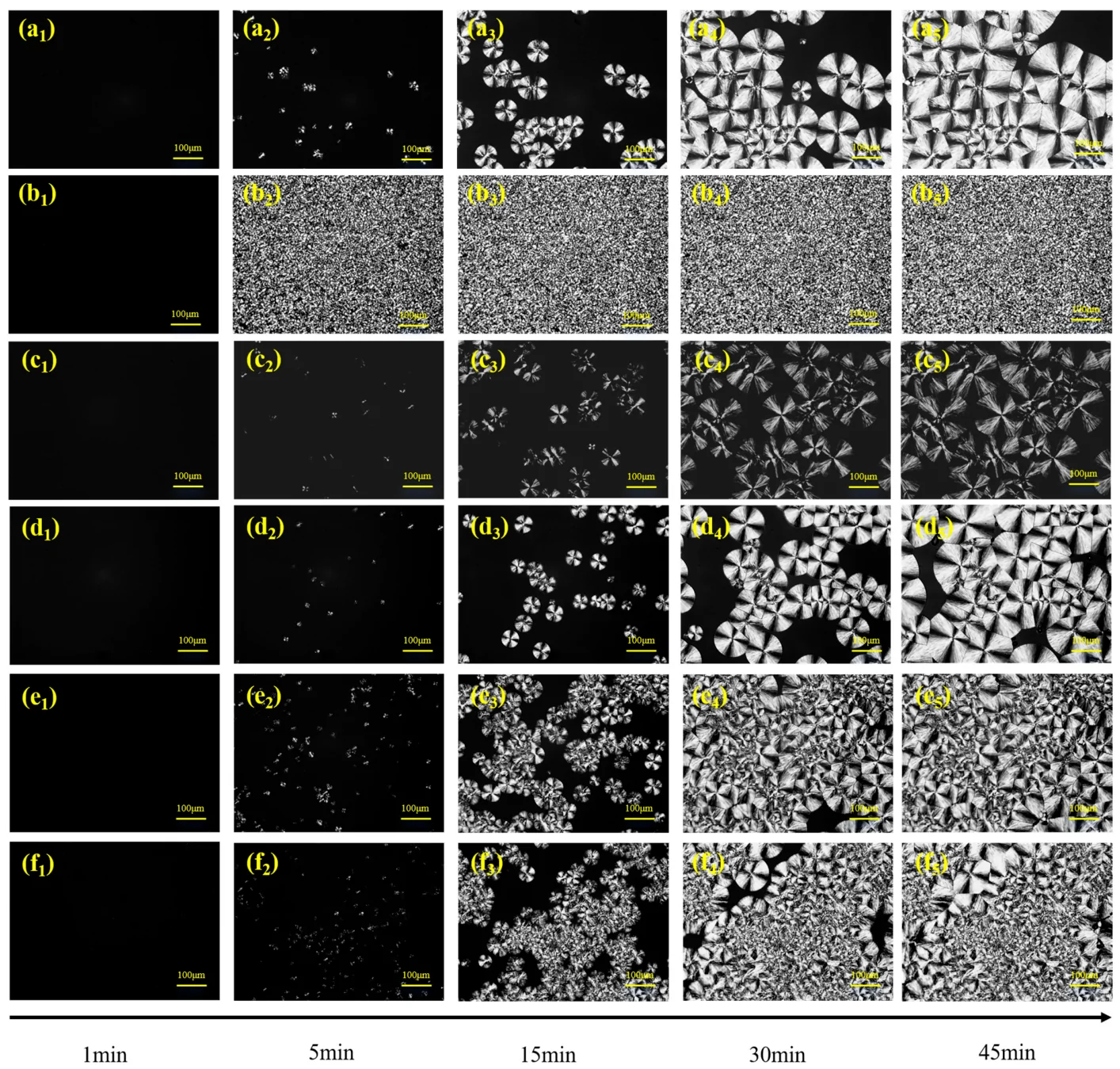
Isothermal crystallization POM images of unfoamed PLA/PPC5 and different unfoamed *PLA/PPC-CE* specimens crystallized at a constant temperature of 130 °C for various durations: (**a_1_**–**a_5_**) PLA, (**b_1_**–**b_5_**) CEPLA, (**c_1_**–**c_5_**) PLA/PPC5, (**d_1_**–**d_5_**) PLA/PPC5-CE0.6, (**e_1_**–**e_5_**) PLA/PPC10-CE0.6 and (**f_1_**–**f_5_**) PLA/PPC15-CE0.6.

An interesting contrast was observed in the cold crystallization region. Pure PLA exhibited a distinct but relatively broad cold crystallization peak, with a cold crystallization enthalpy change (H_cc_) of 29.7 J/g and a cold crystallization temperature (T_cc_) of 110.5 °C. Notably, CEPLA exhibited a highly suppressed cold crystallization peak (T_cc_ = 109.8 °C, H_cc_ = 13.6 J/g), indicating that the branched structure of the chain extender significantly inhibited the cold crystallization rearrangement of PLA segments during heating. Interestingly, a comparison of the PLA/PPC5 and the PLA/PPC5-CE0.6 reveals that the added chain extender plays a key role. The PLA/PPC5 exhibited a relatively broad and flat cold crystallization peak with a low χ_c_ value of only 2.7%. After adding the chain extender, this peak became sharper, and the χ_c_ value slightly rose to 3.3%, suggesting that the chain extender acted as a potential nucleating agent. Nevertheless, the qualitative observation from POM, which showed a significant increase in spherulite number density (Figure 2), more convincingly supported the nucleating role of CE. As the PPC content increased from 5 wt% to 15 wt%, T_cc_ rose significantly from 112.9 °C to 119.5 °C, while the enthalpy of cold crystallization (ΔH_cc_) gradually decreased from 28.6 J/g to 25.7 J/g. The increase in T_cc_ indicated that the addition of PPC inhibited PLA crystallization. On the one hand, the presence of a large amount of the PPC dispersed phase created steric hindrance to the ordered arrangement of PLA molecular chains. On the other hand, the branched structure introduced by the chain extender further restricted the mobility of PLA segments, requiring PLA to reach higher temperatures to achieve sufficient segment mobility to complete crystallization rearrangement. The T_cc_ values of PPC10 and PPC15 were very close, indicating that when the PPC content exceeded 10 wt%, its inhibitory effect on PLA crystallization tended to saturate. The gradual decrease in ΔH_cc_ reflected that the number of crystallizable amorphous PLA segments decreased as the PPC content increased [43].

In terms of melting behavior, after the addition of a chain extender, CEPLA exhibited a single melting peak, indicating that the chain extender can effectively improve PLA crystallization; however, after the addition of PPC, a double melting peak was observed, and a clear trend was noted in the three PLA/PPC-modified samples with different PPC contents. As the PPC content increased from 5 wt% to 15 wt%, the system consistently exhibited dual melting peaks. Of the two endothermic peaks, the one at the higher temperature gradually shifted toward the one at the lower temperature. For example, the PLA/PPC5-CE0.6 sample exhibited dual melting peaks at 163.60 °C and 169.19 °C. However, as the PPC content increased to 15 wt%, the original twin melting peaks nearly merged into a single melting peak. This indicated that the increased content of the PPC dispersed phase, along with the branching structure introduced by the chain extender, disrupted the regularity of the stacking and reduced the thickness of the PLA crystal lamellae. The ΔH_m_ remained between 30 and 32 J/g across the three groups of specimens, showing little variation.

With respect to the relative crystallinity χc, the addition of a chain extender significantly increased the χc of CEPLA, indicating that the formation of a PLA–chain extender–PLA molecular chain structure effectively promoted PLA crystallization. However, upon the addition of PPC, which is an amorphous polymer, it hindered PLA crystallization, resulting in a significant decrease in crystallinity. Second, the addition of CE0.6 elevated it from 2.7% for PLA/PPC5 to 3.3% for PLA/PPC5-CE0.6 due to the nucleating effect. Second, in the CE0.6-modified series, it further increased from 3.3% for PPC5 to 5.1% for PPC10 and 5.9% for PPC15. Although this result appeared to contradict the cold crystallization and cooling crystallization behaviors described above, it actually reflected differences in time scales. Cooling crystallization and heating cold crystallization reflected the crystallization ability of PLA segments under non-isothermal dynamic conditions and were governed by kinetic factors. In contrast, χ_c_ was the total crystallinity calculated by subtracting the enthalpy of cold crystallization from the enthalpy of fusion during the second heating scan, whose value depended on the total amount of crystals accumulated in the specimen prior to reaching the final melting point. As the PPC content increased, the rise in the χ_c_ value can be explained by the crystallization behavior observed during the controlled cooling phase prior to the second heating scan. As shown by the POM observations (Figure 2), an increase in PPC content significantly increased the number of spherulites per unit area; this is because a greater number of PPC dispersed particles provide more heterogeneous nucleation sites for PLA crystallization. At the same time, the branched structure introduced by the chain extender and the physical presence of the PPC dispersed phase created steric hindrance, thereby restricting chain mobility and reducing the spherulite growth rate. The final result was that, although the growth rate was suppressed by kinetic factors, the overall crystallinity was higher, indicating that under the non-isothermal cooling conditions employed, the enhanced nucleation effect outweighed the growth inhibition effect.

The effect of PPC content and the CE0.6 compatibilizer on the thermal properties of the PLA/PPC blend system could be summarized as follows. The addition of CE0.6 effectively improved the interfacial compatibility and acted as a nucleating agent, providing the foundation for increased crystallinity. Simultaneously, the addition of PPC lowered the T_g_ of PLA and enhanced segment mobility. However, the dispersed phase of PPC and the branching structure of the chain extender jointly inhibited the crystallization ability of PLA during cooling and heating, resulting in a weakened cooling crystallization peak, an increase in T_cc_, and a decrease in ΔH_cc_. The final crystallinity, χ_c_, showed an upward trend due to the significant decrease in ΔH_cc_. The PPC15 system, with the highest crystallinity of 5.9%, provided more interfaces between crystalline and amorphous regions during the foaming process, which facilitated the dissolution of CO_2_ and bubble nucleation, and helped enhance the melt strength of the matrix.

### 3.2. POM Observation of PLA/PPC5 and Different PLA/PPC-CE Specimens

To further elucidate the relationship between the microstructure and crystallization behavior of the specimens, isothermal crystallization observations of PLA/PPC/CE specimens with different PPC mass fractions were conducted at 130 °C using a POM. The results were shown in Figure 2a–f.

**Figure 3:**
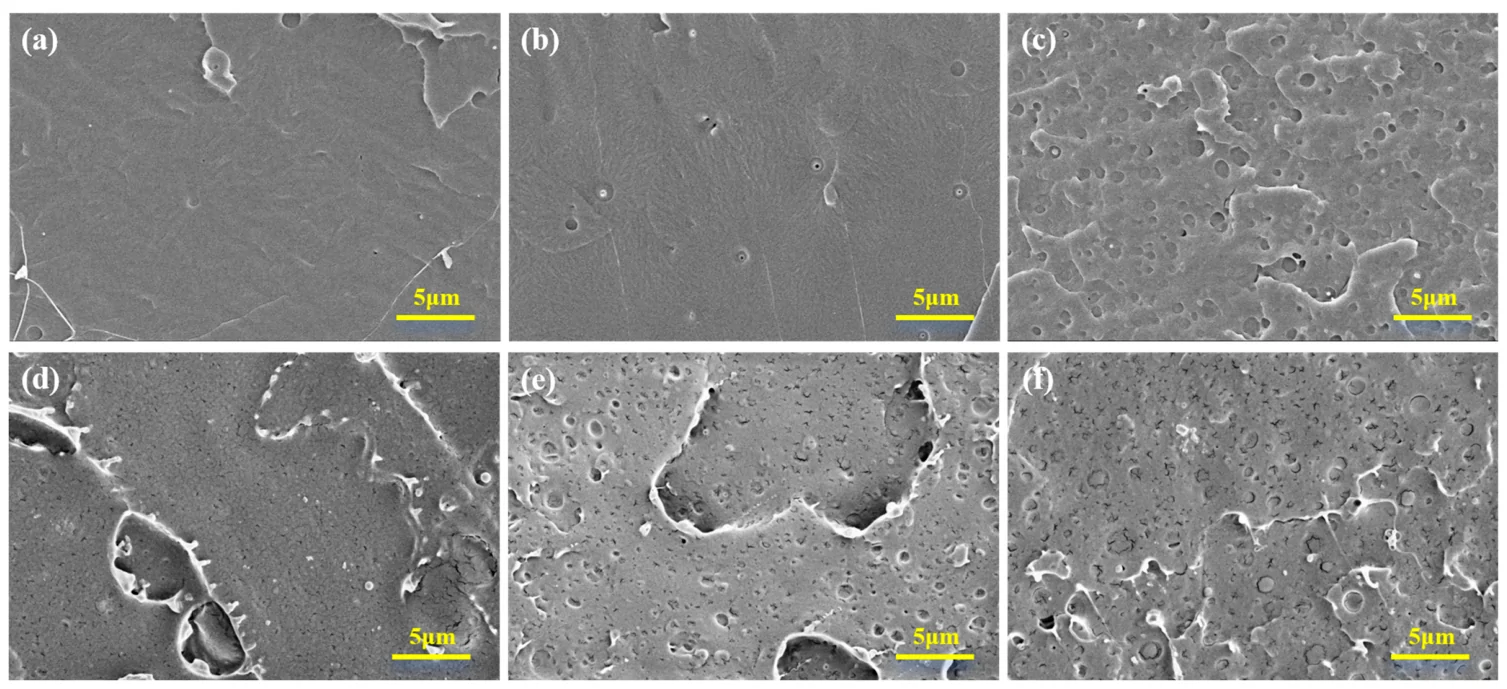
SEM images of (**a**) PLA, (**b**) CEPLA, (**c**) PLA/PPC5, (**d**) PLA/PPC5-CE0.6, (**e**) PLA/PPC10-CE0.6, and (**f**) PLA/PPC15-CE0.6.

First, a comparison of the PLA and CEPLA systems clearly demonstrated the role of chain extenders in promoting the nucleation process. Under the same crystallization time, the CEPLA specimens (Figure 2b_1_–b_5_) exhibited a greater number of fine crystals; however, their crystallinity was also significantly higher. Although this may enhance melt strength, it also hindered the dissolution of carbon dioxide, thereby inhibiting bubble growth and making it difficult to form large-volume bubbles. However, a comparison between PLA and PLA/PPC5 showed that the addition of the amorphous polymer PPC caused the spherulite contours in PLA to become blurred, and its crystallinity was significantly lower than that of CEPLA. In contrast, the PLA/PPC5-CE0.6 samples (Figure 2d_1_–d_5_) exhibited significantly higher spherulite density, finer particle size, and clearer morphology. This morphological difference was consistent with the results of differential scanning calorimetry (DSC), as the addition of the chain extender slightly increased the relative crystallinity from 2.7% to 3.3%. This confirms that, in addition to improving compatibility, the chain extender also acts as an effective heterogeneous nucleating agent, promoting the formation of a large number of small, well-ordered crystals. Combined with cross-sectional scanning electron microscopy (SEM) analysis (Figure 3d), the fine PPC particles were uniformly dispersed in the PLA matrix. These fine dispersed-phase particles acted as heterogeneous nucleating agents for PLA. However, due to their limited number, the nucleation density was low, resulting in larger spherulites.

When the mass fraction of PPC was increased to 10 wt% (Figure 2e_1_–e_5_), under the same isothermal crystallization time, the number of spherulites per unit area increased markedly, and their size decreased significantly. This was attributed to the increased number of PPC dispersed-phase particles, which provided more heterogeneous nucleation sites, promoting the crystallization nucleation of PLA and resulting in higher spherulite density and finer spherulite size. Furthermore, the branched structure introduced by the chain extender KL-E4370 might restrict the movement of PLA molecular chains through physical entanglement, thereby reducing the crystal growth rate and further promoting the formation of smaller spherulites. During the foaming process, these interfacial regions could serve as preferential nucleation sites, facilitating the formation of nanoporous structures with higher cell densities.

When the PPC mass fraction was further increased to 15 wt% (Figure 2f_1_–f_5_), the spherulite morphology underwent significant changes. Compared to the PPC5 and PPC10, the PPC15 exhibited a further increase in the number of spherulites under the same crystallization time. However, the boundaries between spherulites became blurred, with some regions displaying irregular crystalline morphologies, and the characteristic maltesecross of typical spherulites gradually disappeared. This phenomenon indicated that the addition of high-concentration PPC and the effect of chain growth agents in improving the compatibility of PLA and PPC jointly influenced the crystallization behavior of PLA. On the one hand, a large number of PPC particles provided dense nucleation sites, causing spherulites to rapidly compete with one another for growth space, thereby drastically reducing their size. On the other hand, the branched or cross-linked structures formed by the reaction of PPC molecular chains with the chain extender might create steric hindrance to the ordered folding and chain crystallization of PLA, resulting in incomplete crystallization and the formation of a crystal structure with numerous defects.

Combined with cross-sectional SEM analysis (Figure 3f), the larger dispersed-phase particles and their aggregates in the PPC15 acted as nucleation sites during crystallization. These dense nucleation sites enabled spherulites to nucleate in large numbers and grow rapidly within a limited space, ultimately forming fine and incomplete spherulite structures. This crystallization morphology had dual significance for foaming. On the one hand, the numerous fine spherulites provided abundant interfaces between crystalline and amorphous regions, which facilitated CO_2_ dissolution and cell nucleation. On the other hand, the incomplete crystal structure implied the presence of a larger number of amorphous regions, which had higher CO_2_ solubility, further promoting cell growth and facilitating high-ratio foaming [23,36].

### 3.3. Morphological Analysis of the Microstructures of PLA/PPC5 and Various PLA/PPC-CE Specimens

Figure 3 showed SEM images of the fracture surfaces of PLA/PPC specimens with different PPC mass fractions. As shown in Figure 3a–f, all blend systems exhibited a typical “island-in-sea” structure, in which PPC, as the dispersed phase, was uniformly distributed as spherical or near-spherical particles within the PLA continuous matrix phase [44]. No obvious macroscopic phase separation was observed, indicating that the chain extender KL-E4370 effectively facilitated in situ phase expansion at all three ratios.

The comparison between the unmodified PLA/PPC5 (Figure 3c) and the chain-extender-modified PLA/PPC5-CE0.6 specimen (Figure 3d) provided clear evidence of the compatibilizing effect of KL-E4370. As shown in Figure 3c, without the chain extender, the PPC dispersed phase exhibited large, irregular cavities with sizes of approximately 1–2 μm, along with noticeable pull-out traces, indicating poor interfacial adhesion and significant phase separation between PLA and PPC. In sharp contrast, after adding 0.6 wt% KL-E4370 (Figure 3d), the dispersed-phase particles were significantly refined to sub-micron sizes (0.2–1.0 μm) and became tightly embedded in the PLA matrix without visible interfacial voids. This morphological improvement strongly confirmed that the epoxy groups of the chain extender effectively reacted with the terminal carboxyl and hydroxyl groups of PLA and PPC, forming in situ compatibilizers that dramatically reduced interfacial tension and suppressed the coalescence and coarsening of the dispersed phase.

When the mass fraction of PPC was increased to 10 wt% (Figure 3e), the number of dispersed-phase particles increased significantly, with sizes ranging from approximately 0.5 to 1.5 μm. However, no obvious agglomeration or large-scale phase separation structures were observed, and the surface roughness of the cross-section increased. The increased number of PPC dispersed-phase particles provided more heterogeneous nucleation sites for subsequent foaming, while the branched structure introduced by the chain extender enhanced the melt strength, effectively suppressing cell coalescence and collapse, resulting in a substantial increase in VER. When the PPC mass fraction was further increased to 15 wt% (Figure 3f), the dispersed-phase particles grew larger (approximately 1.5–2.0 μm), with some particles of irregular shapes aggregating. The clarity of the phase interfaces increased slightly, indicating that when the PPC content was too high, the chain extender’s capacity to increase volume tended toward saturation. It was worth noting that no obvious pull-out voids were observed in the cross-section, suggesting that even though the interfacial bonding strength decreased, the overall interfacial adhesion remained superior to that of systems without a chain extender, which helped improve the stability of the cell walls during the foaming process. Although some aggregation occurred in the dispersed phase, during the foaming process, these larger PPC particles and their agglomerates actually provided more efficient gas enrichment zones and nucleation interfaces. During the high-temperature, high-pressure CO_2_ saturation process, CO_2_ molecules were preferentially enriched at the dispersed phase/matrix interface; upon pressure relief, a large number of bubbles nucleated simultaneously and grew synergistically, ultimately creating the conditions for achieving high-expansion-ratio nanofoam.

### 3.4. Rheological Properties

**Figure 4:**
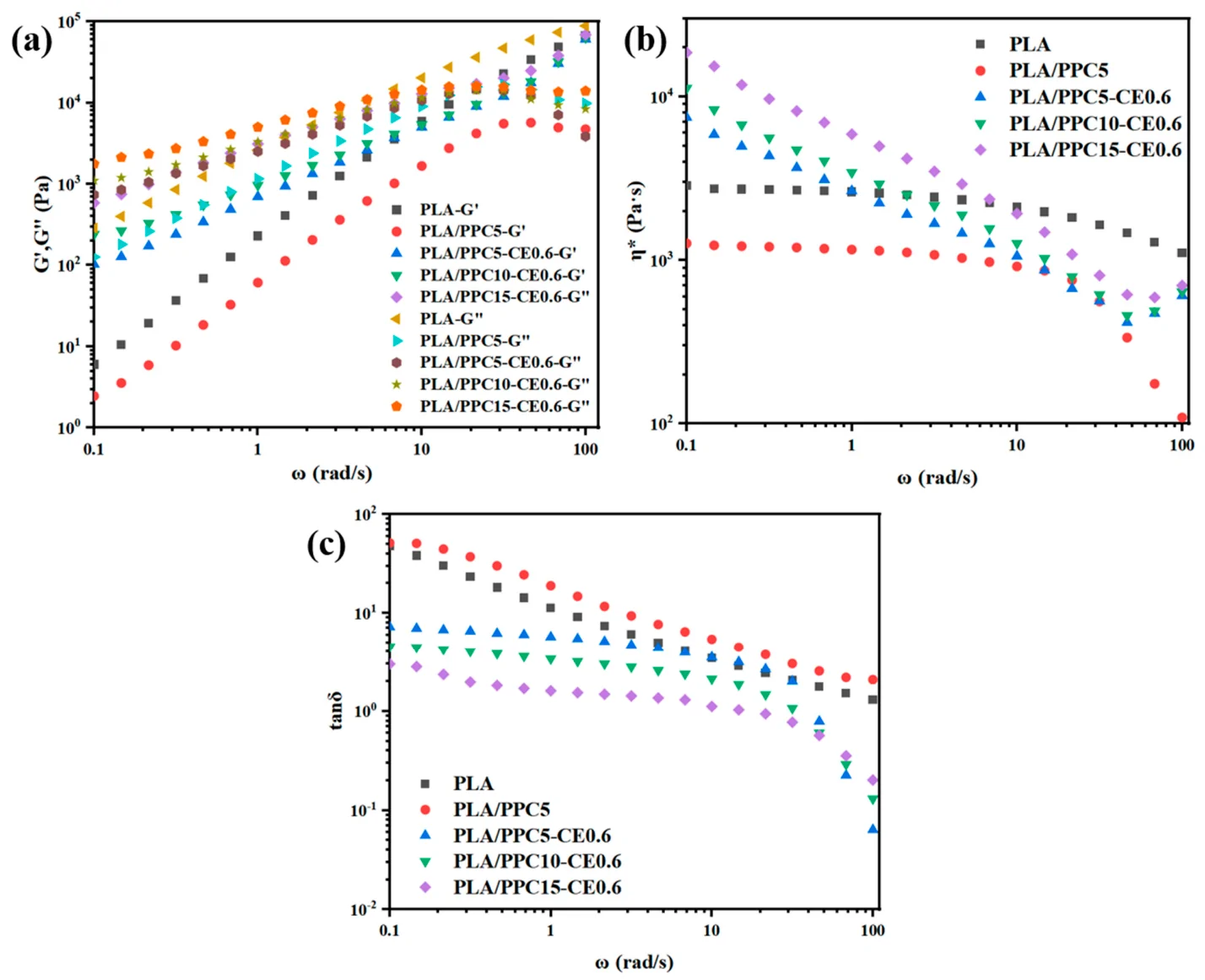
Rheological property curves of PLA and different PLA/PPC-CE specimens: (**a**) G′,G″, (**b**) η*, (**c**) tan δ.

Figure 4a showed the curves of the storage modulus (G′) and loss modulus (G″) of PLA with different PPC contents as a function of angular frequency (ω). In a previous study, Zhou et al. [45] measured the rheological properties of CEPLA. The grades of PLA and chain extenders used in that study were the same as those used in this article. All tested specimens exhibited typical frequency-dependent behavior, with G′ and G″ increasing as ω increased. This is because, at low shear frequencies, the molecular chains of the pure PLA had sufficient time to untangle, resulting in significant molecular chain relaxation; therefore, the material exhibited a lower G′ in the low-frequency range. As ω continued to increase, the relaxation time of the molecular chains far exceeded the deformation time of the pure PLA; the molecular chains could not keep pace with the changes in stress, causing the pure PLA to exhibit hysteresis, and consequently, G′ increased. Significant differences were observed between the unmodified PLA/PPC5 and the specimens modified with a chain extender. In contrast, upon the introduction of the chain extender KL-E4370, G′ and G″ increased significantly at all frequencies, demonstrating that the in situ compatibilization reaction effectively restricted long-range relaxation of the molecular chains through interfacial entanglement. In the low-frequency region, PLA/PPC5-CE0.6 and PLA/PPC10-CE0.6 exhibited viscous-dominant behavior (G″ > G′). However, for the PLA/PPC15-CE0.6, G′ was significantly higher than that of the other formulations. Although its G′ remained slightly lower than its own G″ throughout the low-frequency range, the gap between the two narrowed sharply. The behavior indicated that the specimen tended toward elastic behavior. The phenomenon of significantly enhanced elasticity at a 15 wt% PPC content was most likely due to the high concentration of PPC combining with chain extenders to form a long-chain structure. This structure significantly delayed the long-range relaxation of the molecular chains, resulting in a broadening of the relaxation time spectrum [46].

Figure 4b showed the variation in the complex viscosity (η*) with a function of angular frequency for the five specimens. The unmodified PLA/PPC5 exhibited the lowest complex viscosity and a wide Newtonian plateau at low frequencies, reflecting the typical flow behavior of an incompatible polymer melt with weak molecular interactions and weak melt strength. Conversely, all chain-extender-modified specimens exhibited significant shear thinning behavior across the entire frequency range, which was consistent with the general characteristics of polymer melts with strong molecular entanglement, facilitating processing and molding at higher shear rates [47]. A comparison of specimens with different PPC contents revealed that η* at low frequencies was positively correlated with PPC content: that is, the viscosity of PLA/PPC15-CE0.6 was significantly higher than that of PLA/PPC10-CE0.6 and PLA/PPC5-CE0.6. This increase in viscosity, caused by an increase in the content of fillers or a second component, may be due to a chain-extension reaction of PLA, PPC and the chain extender, resulting in the formation of a long-chain structure of PLA-CE-PPC, which slowed down the molecular chain motion. As shown in Figure S1, both PLA and PPC exhibited distinct carbonyl stretching peaks at approximately 1750 cm^−1^ and characteristic C-O-C absorption peaks in the 1200–1000 cm^−1^ range. Upon addition of CE, the peak shapes and relative intensities of the relevant absorption peaks in the specimen changed, while the characteristic absorption of the epoxy groups in CE was weakened (910, 840 cm^−1^). This indicated that the active groups in the chain extender might have undergone ring-opening reactions with the hydroxyl or carboxyl end groups of PLA and PPC, thereby forming chain-extended or branched structures [45]. It should be noted that the absorption at approximately 2350 cm^−1^ was primarily attributable to background interference from CO_2_ in the air. At the same time, at higher angular frequencies, the three curves exhibited a distinct shear-thinning phenomenon.

Figure 4c showed the variation in the loss tangent (tan δ = G″/G′) with the frequency. tan δ reflected the relative contributions of the viscous and elastic properties of the material. Across the entire test frequency range, as the frequency increased, the time available for segmental motion was insufficient for complete relaxation, leading to an enhanced elastic response of the material. Therefore, the values of tan δ for all specimens decreased monotonically with increasing ω. Remarkably, the unmodified PLA/PPC5 exhibited the highest values of tan δ across the entire frequency sweep, demonstrating a predominantly viscous liquid-like behavior with low elasticity. In contrast, the addition of CE0.6 sharply reduced the values of tan δ, confirming the reinforcing effect of the compatibilizer. Notably, in the low-frequency region, PLA/PPC15-CE0.6 exhibited the lowest value of tan δ, and its curve showed a distinct flattening trend at ω < 1 rad/s. This characteristic of enhanced elasticity at low frequencies typically indicated that the melt of this specimen possessed excellent melt strength, which was highly advantageous for subsequent foaming.

### 3.5. Foaming Properties

**Figure 5:**
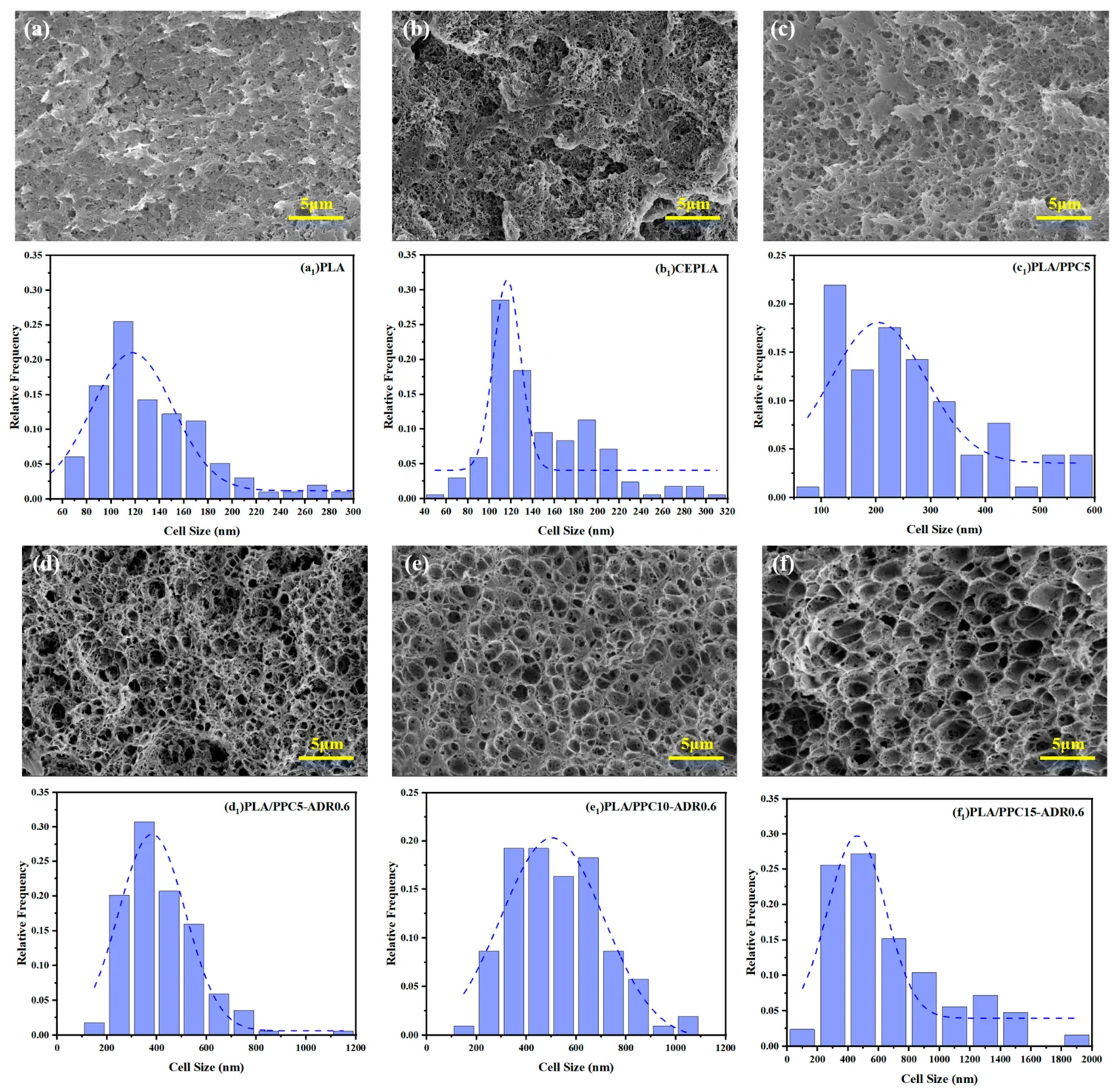
SEM images of PLA and different PLA/PPC-CE0.6 foams at 115 °C and 30 MPa: (**a**) PLA, (**b**) CEPLA, (**c**) PLA/PPC5, (**d**) PLA/PPC5-CE0.6, (**e**) PLA/PPC10-CE0.6, (**f**) PLA/PPC15-CE0.6, and cell distribution of different PLA foams (**a_1_**–**f_1_**).

Figure 5 showed the SEM images and cell size distributions of the PLA/PPC/CE specimens at different PPC mass fractions. The VER, average cell size, and cell density were listed in Table 3.

As shown in Table 3, PLA, CEPLA, and PLA/PPC5 exhibited similar VER, but there are significant differences in their average cell sizes. A comparison of the cell size distribution diagrams for PLA and CEPLA revealed that, although the average cell size of CEPLA was slightly larger than that of PLA, a larger proportion of its cells fell within the range of 100 nm to 200 nm, and its cell density was also higher. This was because the branched/cross-linked structure formed by chain extension not only enhanced melt strength but also provided a large number of heterogeneous nucleation sites. Furthermore, the addition of PPC increased the average cell size. This was because PPC effectively increased the solubility of CO_2_ while simultaneously reducing the melt viscosity and modulus of the material, allowing cells to grow and ultimately form larger cells.

The foaming results showed that the PLA/PPC-CE0.6 specimen produced the narrowest average cell size distribution, concentrated around 400 nm, with the smallest mean cell size of 413 nm and a cell density of 5.2 × 10^12^ cells/cm^3^. However, the VER was only 2.7, the lowest among the three kinds of specimens. This suggested that although the fine dispersed phase and intact spherulite structure provided uniform nucleation sites (Figure 3a_1_–a_3_) and resulted in size-uniform nanoporous cells, the total number of nucleation sites was insufficient. Furthermore, the intact spherulite structure implied a high proportion of crystalline regions and a relatively low proportion of amorphous regions, which limited CO_2_ solubility and ultimately led to insufficient cell growth and a low foaming VER.

Upon raising PPC loading to 10 wt%, the foam attained a VER of 5.1 alongside a mean cell size of 527 nm, even though its cell density was marginally lower compared with the 5 wt% PPC specimen. The cell size distribution was slightly broader than that of the PPC5 specimen but remained primarily concentrated in the 400–700 nm range. These results indicated that the increased number of PPC dispersed-phase particles caused more heterogeneous nucleation sites, while the melt strength enhanced by the chain extender effectively suppressed cell coalescence and collapse, leading to a substantial increase in the VER. However, the slight decrease in cell density might be attributed to the fact that, as the PPC content increased, the size distribution of the dispersed phase broadened. Some larger PPC particles tended to form a few larger cells rather than a large number of small cells during the foaming process, resulting in a slight decrease in cell density. Overall, the PPC10 system achieved a good balance between VER and cell size.

At a PPC content of 15 wt%, the highest VER of 7.3 times and the highest cell density of 1.1 × 10^13^ cells/cm^3^ were achieved, with the mean cell size of 660 nm. It was worth noting that although the average cell size was the largest among the kinds of specimens, the specimen exhibited the highest cell density and the widest size distribution, ranging from approximately 200 nm to approximately 1600 nm, with the distribution histogram showing distinct characteristics of a broad distribution. However, 90% of the cells fell approximately within the nanoscale range; this might be because the large amount of dispersed PPC and its aggregates in the PPC15 provided heterogeneous nucleation sites far exceeding those in the PPC5 and PPC10. At the same time, the fine and incomplete spherulite structure observed in POM provided abundant interfaces between crystalline and amorphous regions. These interface areas exhibited higher affinity for CO_2_, further promoting heterogeneous nucleation of the cells. The synergistic effect of these two factors enabled the preparation of a high-expansion-ratio nanofoam with an VER of 7.3 times and a mean cell size of 660 nm.

**Figure 6:**
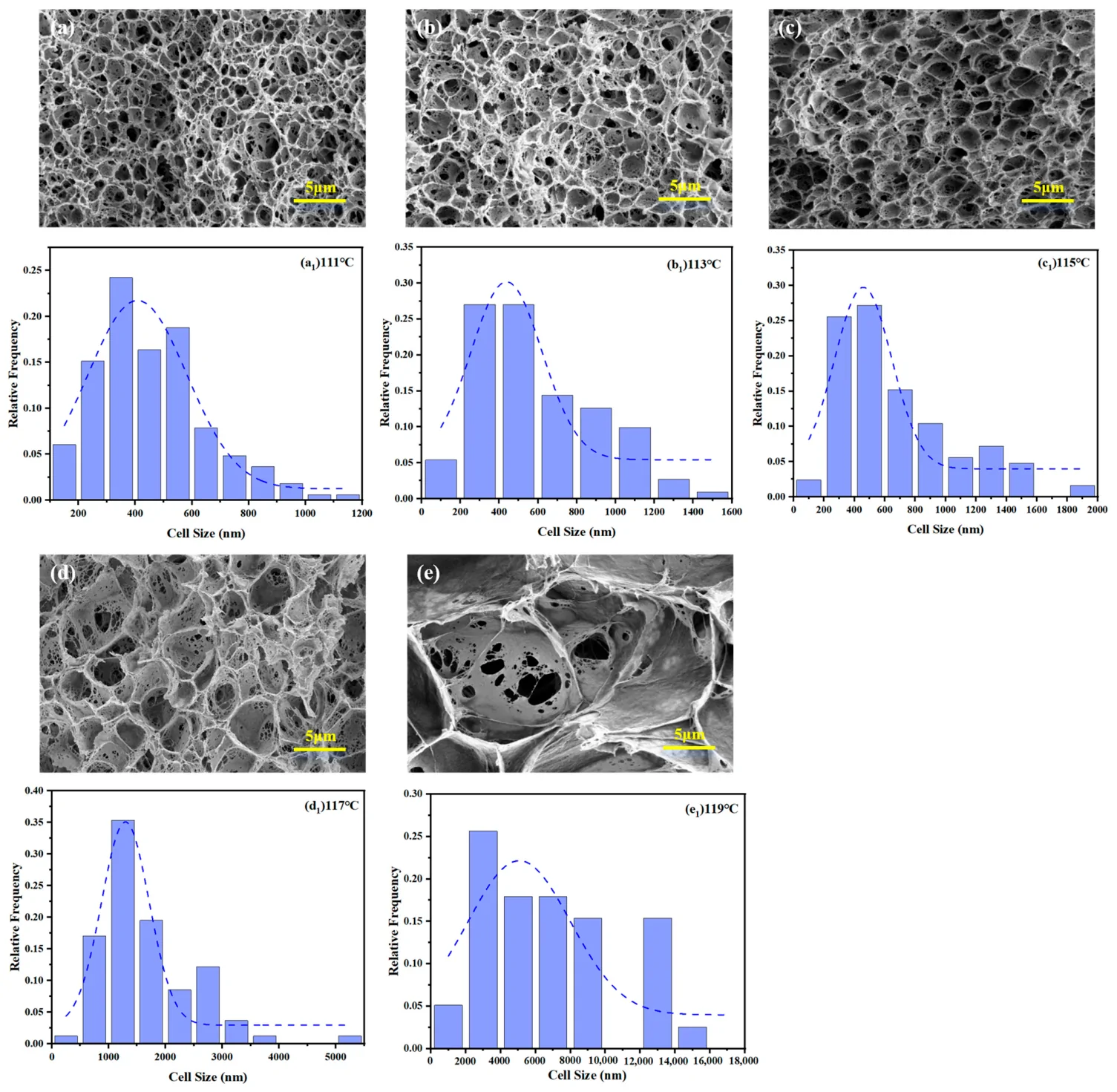
SEM images of PLA/PPC15-CE0.6 foam at 30 MPa and different temperature conditions: (**a**) 111 °C, (**b**) 113 °C, (**c**) 115 °C, (**d**) 117 °C, (**e**) 119 °C, and the cell distribution of PLA-PPC15 foam under different temperature conditions (**a_1_**–**e_1_**).

Based on the determination that PLA/PPC15-CE0.6 was the optimal formulation, the effect of the foaming temperature on the cell structure and foaming performance of this system was further investigated. Figure 6 showed the SEM images of this specimen at varying foaming temperatures (111, 113, 115, 117, and 119 °C). The corresponding cell size distribution histograms were shown in Figure 6a_1_–e_1_, and the VER, average cell size, and cell density were listed in Table 4.

At a foaming temperature of 111 °C, the foaming VER was only 3.4 times, the average cell size was 466 nm, and the cell density was 6.6 × 10^12^ cells/cm^3^. At this temperature, the melt strength of the PLA matrix was excessively high, and the mobility of the molecular segments was insufficient, resulting in low CO_2_ diffusion rates and solubility within the matrix. After bubble nucleation, it was difficult to obtain a sufficient gas supply for effective growth. Therefore, the bubble size was the smallest, but the VER was also the lowest, and the bubble size distribution was the most concentrated. Upon elevating the temperature to 113 °C, the VER reached 4.8 times, the mean cell size grew to 594 nm, and the cell density dropped marginally to 5.0 × 10^12^ cells/cm^3^. With rising foaming temperature, the mobility of the PLA segments enhanced, the CO_2_ diffusion rate increased, cell growth became more thorough, and the VER improved significantly. The slight decrease in cell density was attributed to limited coalescence of some adjacent cells during the growth process.

The most critical turning point in this study was 115 °C. The VER surged to 7.3 times, the average cell size reached 660 nm, and the cell density reached 1.1 × 10^13^ cells/cm^3^. This cell density was the highest value observed under all temperature conditions, more than doubling that at 113 °C. This significant improvement indicated that at 115 °C, the system achieved a synergistic optimum in nucleation efficiency and growth capacity. On the one hand, the temperature was high enough to provide the PLA matrix with suitable segment mobility and CO_2_ diffusion rates, ensuring sufficient bubble growth. On the other hand, the melt strength of the matrix remained sufficient to effectively suppress bubble coalescence and collapse, allowing a large number of nucleated bubbles to grow independently and stably. It was particularly worth noting that the cell density exhibited a sharp increase at this temperature, indicating that a large number of cells nucleated simultaneously at the moment of pressure release at 115 °C. This temperature was therefore the optimal foaming temperature for this specimen.

At the foaming temperature of 117 °C, the VER further attained 9.3 times. In contrast, the mean cell size surged to 1672 nm, while cell density plummeted to 7.9 × 10^11^ cells/cm^3^, representing a reduction of more than one order of magnitude compared with 115 °C samples. The cell size distribution broadened significantly and shifted overall to the 1000–3000 nm range. This change indicated that 117 °C exceeded the critical temperature at which this specimen could maintain a nanoporous structure. At this temperature, the PLA matrix became excessively softened due to the combined effects of rising temperature and the CO_2_ plasticizing effect, causing a sharp decline in melt strength. This led to severe cell coalescence, resulting in a reduction in cell count and a dramatic increase in cell size [48]. When the temperature was further increased to 119 °C, the VER reached as high as 15.6 times. However, the average cell size expanded dramatically to 6687 nm, and the cell density further decreased to 2.8 × 10^10^ cells/cm^3^. This cell density represented a decrease of nearly three orders of magnitude compared to that at 115 °C, and the cell size dispersion broadened significantly, exhibiting typical open-cell and coalescence characteristics. At this temperature, the PLA matrix softened excessively, and the melt strength was insufficient to maintain the stability of the cell walls. In consequence, a large number of cell walls ruptured, forming an open-cell structure and giant cells. Although the VER was the highest, the cell structure was severely degraded.

### 3.6. Foaming Mechanism

**Figure 7:**
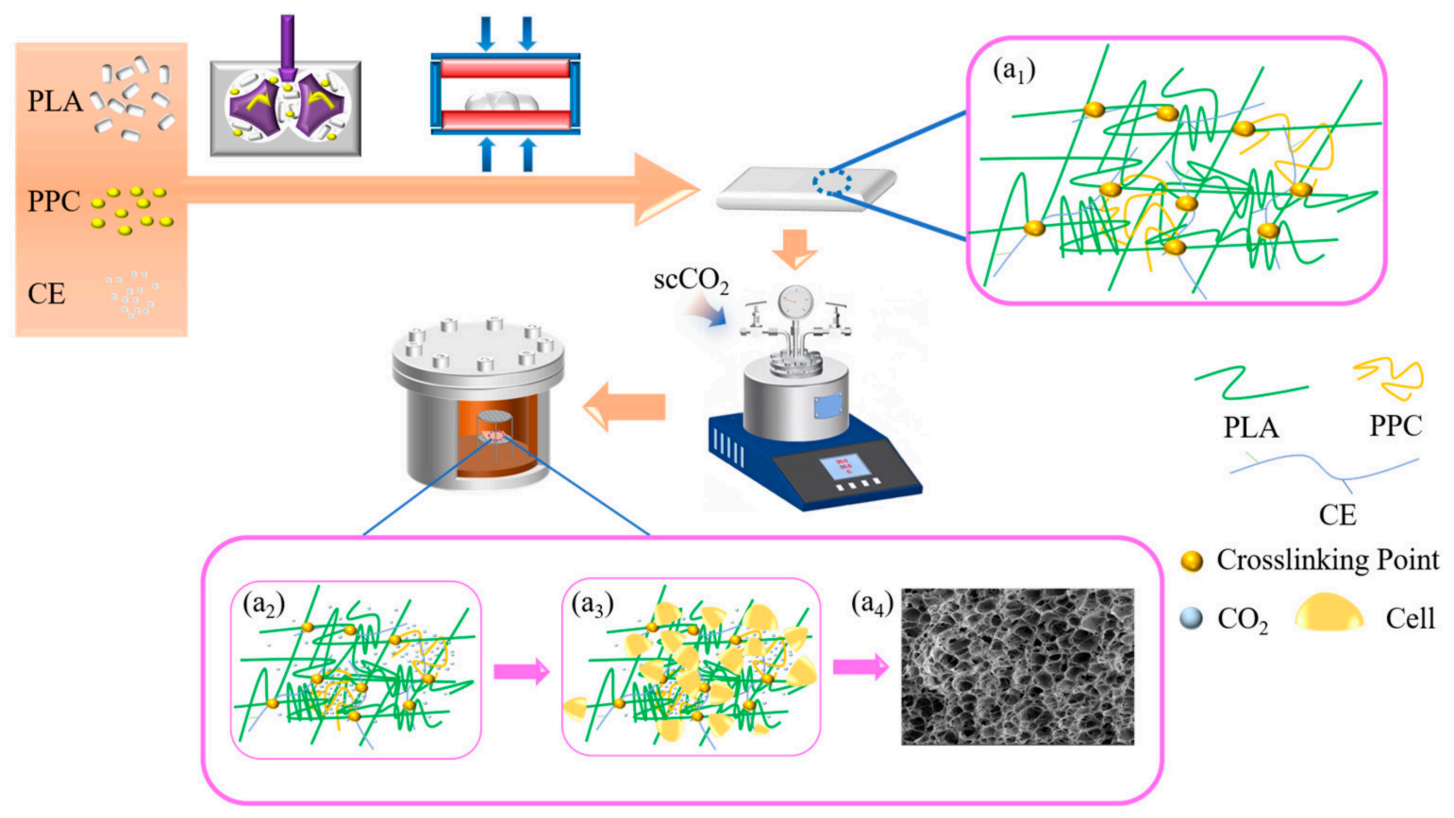
Foaming mechanism schematic of PLA/PPC-CE0.6 specimens (**a_1_**–**a_4_**).

The formation of high-VER nanofoam structures in the PLA/PPC-CE0.6 system was the result of the coordinated action of four factors. Firstly, the epoxy groups on the chain of the chain extender KL-E4370 underwent ring-opening addition covalently couple with the terminal carboxyl and hydroxyl groups of PLA and the terminal hydroxyl groups of PPC during the melt blending process, thereby generating a PLA–chain extender–PPC block copolymer in situ (Figure 7a_1_). This reaction not only achieved chain extension and viscosity increase, but also reduced the interfacial tension, enabling the uniform distribution of the PPC dispersed phase and acting as heterogeneous nucleation sites and inhibiting the thermal degradation of PLA, providing suitable melt strength and interfacial properties for foaming. On this basis, a large number of PPC/PLA interfaces served as main nucleation sites, significantly reducing the activation free-energy barrier associated with CO_2_ heterogeneous nucleation, and the 5.9% crystallinity revealed by DSC indicated the presence of a large number of fine and imperfect crystalline regions. The interface between the crystalline region and the amorphous region, together with the PPC/PLA interface, formed a nucleation site system (Figure 7a_3_), which collaboratively achieved a cell density of 1.1 × 10^13^ cells/cm^3^ at 115 °C (Figure 7a_4_), which was approximately twice as high as the PPC5 and PPC10. At the same time, these crystalline regions acted as physical cross-linking points, further enhancing the stability of the cell wall and effectively inhibiting cell merging. The selection of the foaming temperature was a key process parameter that determined the final cell structure. The T_g_ of the PLA/PPC15-CE0.6 was 58.9 °C and the T_cc_ was 119.5 °C. At 115 °C, which was exactly within the appropriate viscosity range between T_g_ and T_cc_, the movement ability of the PLA chain segments was sufficient to support the rapid diffusion of CO_2_ and the full growth of cells, while the residual crystalline regions and the branched structure of the chain extender jointly maintained sufficient melt strength to inhibit excessive growth and merging of cells. The foaming rate and cell density showed an order-of-magnitude jump at this temperature. In addition, a large number of PPC/PLA interfaces and the interface between the crystalline region and the amorphous region provided gas dissolution sites during the CO_2_ saturation stage, allowing the system to dissolve more CO_2_ (Figure 7a_2_), providing a sufficient gas source for bubble growth [33,34,35]. This was another key factor for the 7.3-fold high-VER foaming of the PPC15.

### 3.7. Mechanical Property Measurement

**Figure 8:**
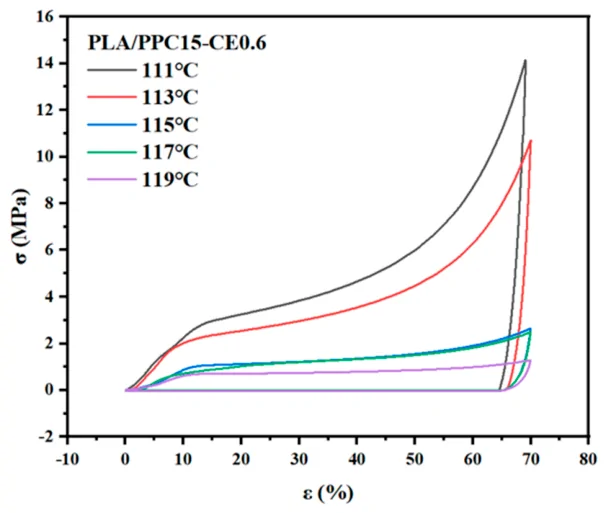
Compression test results of PLA/PPC15-CE0.6 foam at different temperatures.

Figure 8 showed the compressive strength of PLA/PPC15-CE0.6 foams at 70% deformation under different foaming temperatures. Corresponding foaming performance parameters were listed in Table 4 for comparison.

Overall, the compressive strength decreased monotonically as the foaming temperature increased. The specimen foamed at 111 °C exhibited the highest compressive strength of 14.0 MPa, whereas the specimen foamed at 119 °C had a compressive strength of only 0.5 MPa, representing a decrease of more than 96%. The fundamental reason for the decrease in compressive strength upon elevating the foaming temperature stemmed from the significant increase in the foaming VER and the enlargement of the average cell size. The proportion of the polymer backbone per unit volume continuously decreased, and the cell wall thickness became smaller, resulting in a corresponding decline in the load-bearing capacity of the foam when subjected to compressive loads.

A correlation analysis between compressive strength and the corresponding cell-structure parameters provided a deeper understanding of the structure–property relationship. The specimen foamed at 111 °C had the smallest cell size (466 nm) and the highest cell density (6.5 × 10^12^ cells/cm^3^). However, because the foaming VER was only 3.4 times and the cell wall thickness was relatively large, it exhibited the highest compressive strength of 14.0 MPa. For the specimen foamed at 113 °C, the VER increased to 4.8 times, the cell size increased to 594 nm, and the compressive strength decreased to 11.5 MPa. For the specimen foamed at 115 °C, with the VER jumping to 7.3 times and the cell size increasing to 660 nm, the compressive strength was 2.7 MPa, a significant decline compared to the specimen foamed at 113 °C. This indicated that when the VER exceeded a certain critical value, the compressive strength decreased dramatically, which might be related to the significant thinning of the cell walls and the increased connectivity of the cell structure. Of particular note was that the compressive strength of the 115 °C specimen also decreased significantly as the cell wall got thinned.

With a further increase in foaming temperature to 117 °C, the foaming VER reached 9.3; concomitantly, the mean cell size rose sharply to 1672 nm, the cell density declined drastically to 7.9 × 10^11^ cells/cm^3^, and the compressive strength further decreased to 2.1 MPa. Although this represented a decrease of only 0.6 MPa compared to the 115 °C specimen, the cell structure had already undergone a qualitative change. Although the specimen at 119 °C achieved a foaming VER as high as 15.6 times, its compressive strength dropped sharply to 0.5 MPa due to severe deterioration of the cell structure, virtually losing its load-bearing capacity and rendering it completely useless for practical applications.

## 4. Conclusions

This study successfully fabricated nanocellular foams of PLA/PPC featuring high VER using scCO_2_ foaming technology by introducing the multifunctional epoxy-based chain extender KL-E4370 into the PLA/PPC, systematically elucidating the foaming mechanism and the relationship of structure–property. The experimental results showed that the chain extender effectively underwent reaction with the terminal carboxyl and hydroxyl groups of PLA and PPC through ring-opening of its cyclic functional moieties, generating a PLA–extender–PPC copolymer in situ, which significantly enhanced melt strength, reduced interfacial tension, and promoted uniform dispersion of the PPC phase. Notably, the PLA/PPC15-CE0.6 formulation achieved optimal cell morphology at a foaming temperature of 115 °C, with the VER of 7.3 times, the mean cell size of 660 nm, and the cell density as high as 1.1 × 10^13^ cells/cm^3^. Moreover, the foaming temperature exhibited a critical threshold effect: when the temperature rose to 117 °C or above, excessive softening of the matrix triggered cell coalescence, causing the cellular structure to destabilize from the nanoscale to the microscale and resulting in a more than one-order-of-magnitude drop in cell density. The specimen foamed at 115 °C retained a compressive strength of 2.7 MPa under 70% strain, demonstrating an excellent balance between high VER and mechanical integrity. This study revealed a synergistic foaming mechanism involving chain-extender-induced enhancement of melt strength, multi-interface heterogeneous nucleation, and critical temperature-controlled nucleation–growth–stabilization equilibrium, providing both theoretical insights and practical guidance for developing high-performance, highly VER bio-based polymer nanocellular foams.

## Figures and Tables

**Table 1:** Formulations for various PLA test specimens.

Specimens	PLA	CEPLA	PLA/PPC5	PLA/PPC5-CE0.6	PLA/PPC10-CE0.6	PLA/PPC15-CE0.6
PLA (wt%)	100	99.4	95	94.4	89.4	84.4
PPC (wt%)	0	0	5	5.0	10.0	15.0
CE (wt%)	0	0.6	0	0.6	0.6	0.6

**Table 2:** Thermal property parameters of different PLA/PPC-CE specimens.

Specimens	T_g_/(°C)	T_m1_/(°C)	T_m2_/(°C)	∆H_m_/(J/g)	T_cc_/(°C)	∆H_cc_/(J/g)	χ_c_/%
PLA	60.7 ± 0.2	163.3 ± 0.3	169.4 ± 0.2	34.5 ± 0.1	110.5 ± 0.1	29.7 ± 0.2	5.1 ± 0.2
CEPLA	61.2 ± 0.1	-	168.2 ± 0.1	33.9 ± 0.2	109.8 ± 0.2	13.6 ± 0.1	21.8 ± 0.1
PLA/PPC5	59.8 ± 0.2	164.5 ± 0.1	170.2 ± 0.2	31.0 ± 0.1	111.9 ± 0.1	28.6 ± 0.1	2.7 ± 0.1
PLA/PPC5-CE0.6	60.1 ± 0.3	163.6 ± 0.2	169.2 ± 0.2	31.7 ± 0.1	112.9 ± 0.1	28.7 ± 0.2	3.3 ± 0.1
PLA/PPC10-CE0.6	59.2 ± 0.2	165.0 ± 0.3	168.7 ± 0.1	32.5 ± 0.2	119.9 ± 0.2	28.2 ± 0.1	5.1 ± 0.2
PLA/PPC15-CE0.6	58.9 ± 0.1	165.4 ± 0.2	168.3 ± 0.3	30.3 ± 0.1	119.5 ± 0.1	25.7 ± 0.1	5.9 ± 0.2

**Table 3:** Foaming parameters of PLA and different PLA/PPC-CE0.6 foams.

Foam Name	VER	Cell Size (nm)	Cell Density (Cells/cm^3^)
PLA	2.0 ± 0.1	128 ± 28	1.0 × 10^13^
CEPLA	2.1 ± 0.1	144 ± 34	3.3 × 10^13^
PLA/PPC5	2.1 ± 0.1	263 ± 72	9.6 × 10^12^
PLA/PPC5-CE0.6	2.7 ± 0.3	413 ± 124	5.2 × 10^12^
PLA/PPC10-CE0.6	5.1 ± 0.2	527 ± 187	4.8 × 10^12^
PLA/PPC15-CE0.6	7.3 ± 0.2	660 ± 395	1.1 × 10^13^

**Table 4:** Foaming parameters of PLA/PPC15-CE0.6 foam at different foaming temperatures.

Foaming Temperature (°C)	VER	Cell Size (nm)	Cell Density (Cells/cm^3^)	Cell Wall Thickness (nm)
111	3.4 ± 0.1	466 ± 184	6.6 × 10^12^	113 ± 25
113	4.8 ± 0.3	594 ± 287	5.0 × 10^12^	94 ± 22
115	7.3 ± 0.2	660 ± 395	1.1 × 10^13^	66 ± 13
117	9.3 ± 0.4	1672 ± 808	7.9 × 10^11^	61 ± 14
119	15.6 ± 0.3	6687 ± 3765	2.8 × 10^10^	158 ± 40

## Data Availability

The original contributions presented in this study are included in the article/Supplementary Material. Further inquiries can be directed to the corresponding author.

## Supplementary Materials

The following supporting information can be downloaded at https://www.mdpi.com/article/10.3390/polym18182273/s1, Figure S1: FTIR spectrum of PLA, PPC, KL-E4370 and blends; Figure S2: SEM images of different PLA/PPC15-CE foams at 30MPa: (a) PLA/PPC15-CE0.3, (b) PLA/PPC15-CE0.6, and (c) PLA/PPC15-CE0.9; Figure S3: Chemical structure of KL-E4370; Table S1: Foaming parameters of PLA and different PLA/PPC-CE foams.
