# Supplementary material for: Ultra-High-Expansion-Ratio Nanocellular Semicrystalline Polymer Foams: Preparation of Chain-Extender-Mediated PLA/PPC Foams via Supercritical CO2
Source: Polymers (Basel). 2026 Sep 17;18(18):2273. doi: 10.3390/polym18182273 (PMC13611450; doi:10.3390/polym18182273)
Supplement: Supplementary file 1 [file polymers-18-02273-s001.zip › polymers-4548464-supplementary.pdf]

# Supporting information

## Ultra-High-Expansion-Ratio Nanocellular Semicrystalline Polymer Foams: Preparation of Chain-Extender-Mediated PLA/PPC Foams via Supercritical $CO_2$

Xubo Zhao [1], Xuejun Liu [2,*], Fengrui Hu [1],Hongfu Zhou [1], and Yafeng Deng [2]

1 Key Laboratory of Processing and Application of Polymeric Foams of China National Light Industry Council, School of Advanced Materials and Future Technology, Beijing Technology and Business University, Beijing 100048, China; 2431042106@st.btbu.edu.cn (X.Z.); 17507561319@163.com (F.H.); zhouhongfu@th.btbu.edu.cn (H.Z.)

2 School of Computer and Artificial Intelligence, Beijing Technology and Business University, Beijing 100048 , China; dengyafeng@th.btbu.edu.cn

* Correspondence: liuxj@th.btbu.edu.cn

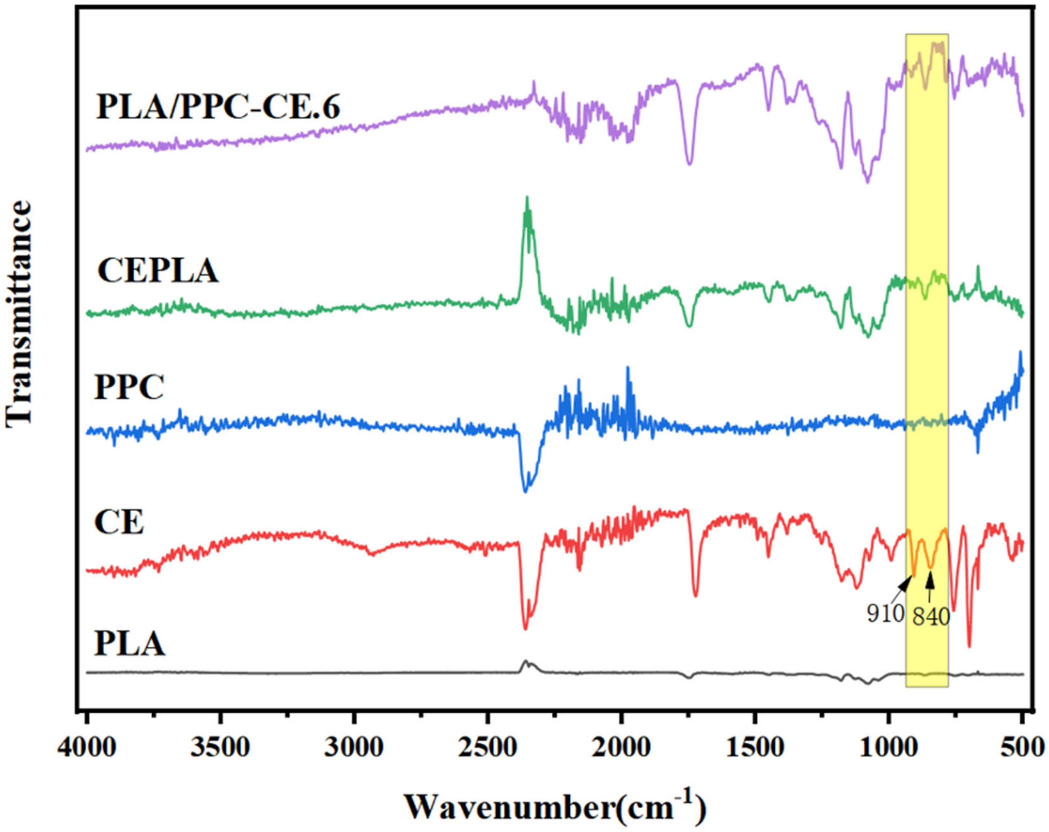


**Figure S1.** FTIR spectrum of PLA, PPC, KL-E4370 and blends.

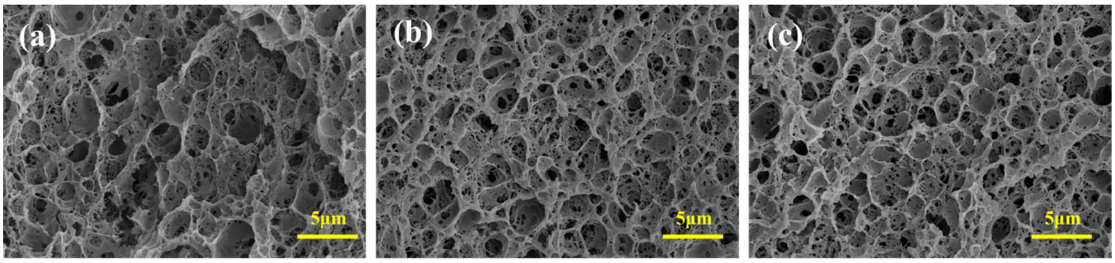


**Figure S2.** SEM images of different PLA/PPC15-CE foams at 30MPa: (a) PLA/PPC15-CE0.3, (b) PLA/PPC15-CE0.6, and (c) PLA/PPC15-CE0.9.

**Table S1**. Foaming parameters of PLA and different PLA/PPC-CE foams.

| Samples | VER | Cell size(nm) | Cell density (cells/cm$^3$) |
|---|---|---|---|
| PLA/PPC15-CE0.3 | 3.5 | 1136 | $6.5\times10^{11}$ |
| PLA/PPC15-CE0.6 | 4.1 | 754 | $2.8\times10^{12}$ |
| PLA/PPC15-CE0.9 | 3.7 | 730 | $3.1\times10^{12}$ |

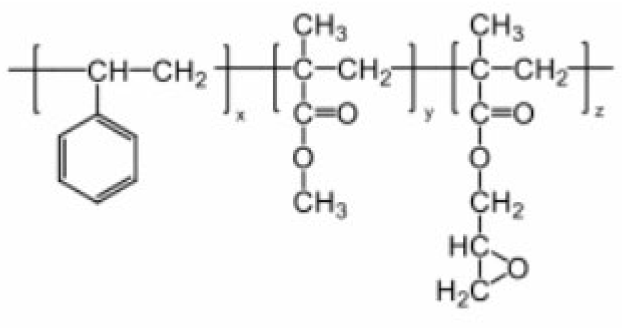


**Figure S3.** Chemical structure of KL-E4370.
